# Hypoxia increases KIAA1199/CEMIP expression and enhances cell migration in pancreatic cancer

**DOI:** 10.1038/s41598-021-97752-z

**Published:** 2021-09-14

**Authors:** Takuya Oba, Norihiro Sato, Yasuhiro Adachi, Takao Amaike, Yuzan Kudo, Atsuhiro Koga, Shiro Kohi, Keiji Hirata

**Affiliations:** 1 Department of Surgery 1, School of Medicine, University of Occupational and Environmental Health, Kitakyushu, 807-8555 Japan; 2 Department of Surgery, Kyushu Rosai Hospital, Kitakyushu, Japan

**Keywords:** Biochemistry, Cancer

## Abstract

Pancreatic ductal adenocarcinoma (PDAC) is characterised by dense desmoplasia and hypoxic microenvironment. Our previous reports demonstrated that hyaluronan (HA), especially low-molecular-weight HA, provides a favourable microenvironment for PDAC progression. However, the effect of hypoxia on HA metabolism remains unknown. Using quantitative real-time RT-PCR and western blot analysis, we analysed the changes in the expression of HA-synthesizing enzymes (HAS2 and HAS3) and HA-degrading enzymes (HYAL1, KIAA1199/CEMIP) in PDAC cell lines under hypoxic conditions. Hypoxia increased the mRNA and protein expression of KIAA1199, whereas it decreased HYAL1 expression. The expression of HAS3 was increased and HAS2 remained unchanged in response to hypoxia. The effect of KIAA1199 on hypoxia-induced cell migration was determined using a transwell migration assay and small-interfering RNA (siRNA). Hypoxia enhanced the migratory ability of PDAC cells, which was inhibited by KIAA1199 knockdown. We also used immunohistochemistry to analyse the protein expression of hypoxia inducible factor (HIF) 1α and KIAA1199 in PDAC tissues. There was a significant immunohistochemically positive correlation between KIAA1199 and HIF1α. These findings suggest that hypoxia-induced KIAA1199 expression may contribute to enhanced motility in PDAC.

## Introduction

Pancreatic ductal adenocarcinoma (PDAC) is one of the deadliest diseases worldwide, with the lowest survival rate among all cancer types^1^. PDAC is characterised by a dense desmoplastic stroma enriched with hyaluronan (HA)^2,3^.

HA is produced by HA-synthesizing enzymes (HASs) and is degraded into smaller fragments by hyaluronidases (HYALs)^4–6^. HA plays a critical role in a variety of malignant behaviours including cell proliferation, migration, invasion, metastasis, angiogenesis, and resistance to drug delivery^7–12^. In particular, low-molecular-weight HA (LMW-HA, 100 kDa or less) has been suggested to be essential in cancer progression^13,14^.

KIAA1199 has been newly identified as an enzyme involved in HA degradation. Previous studies have shown that KIAA1199 is overexpressed in various cancers, and is associated with an aggressive phenotype^15–19^. Notably, KIAA1199 enhances the motility of colon cancer cells and is therefore called the cell migration-inducing protein (CEMIP). We have also demonstrated previously that KIAA1199 expression correlates with poor prognosis in PDAC, and that forced expression of KIAA1199 increases the migration of PDAC cells^20^.

Hypoxia is one of the most common features in the tumour microenvironment^21^. Previous reports have shown that the average oxygenation in PDAC was markedly lower than that in normal tissue (the median pO_2_ of tumours was between 0 and 5.3 mmHg, whereas the median pO_2_ of normal adjacent tissue was between 9.3 and 92.7 mmHg^22^. Tumour growth results in hypoxia and hypoxia itself plays an important role in accelerating tumour progression, malignancy, and treatment resistance. Hypoxia signalling is mediated by hypoxia-inducible factors (HIFs), which are stabilised under hypoxic conditions^23^.

Increased HIF-1α and/or HIF-2α levels in biopsied specimens are associated with an increased mortality risk in some cancer types including PDAC^24^. Although many signalling pathways including mitogen-activated protein kinase (MAPK) signalling^25^, are known to be altered by hypoxic conditions in PDAC cells^25^, the effect of hypoxia on HA metabolism is unknown. In the present study, we investigated the relationship between hypoxia and HA metabolism in PDAC cells.

## Materials and methods

### Cell culture and reagents

Two PDAC cell lines, BxPC3 and Panc-1 (American Type Culture Collection, Manassas, VA, USA) were used in this study. PDAC cell lines were maintained in RPMI-1640 medium (Life Technologies, Grand Island, NY, USA) supplemented with 10% foetal bovine serum (FBS) (Life Technologies) and 1% streptomycin and penicillin (Life Technologies) in a 5% CO_2_ incubator at 37 °C. For hypoxic conditions, the cells were exposed to 1% O_2_ using a low oxygen culture kit (BIONIX low-oxygen culture kit, manufactured by Sugiyamagen Co., Ltd.) for 6–48 h. All experiments were repeated three times.

### Quantitative real-time RT-PCR

Total RNA was isolated from cell lines using the RNeasy Mini Kit (QIAGEN GmbH, Hilden, Germany) according to the manufacturer’s protocol. First strand cDNA was synthesised from 1.0 μg of total RNA using a SuperScript VILO cDNA synthesis Kit and Master Mix (Thermo Fisher Scientific Inc., Waltham, MA, USA). Real-time mRNA expression analysis of HA-related genes (HAS2, HAS3, HYAL1, and KIAA1199), carbonic anhydrase 9 (CA9) and VEGF as a positive internal control and a housekeeping gene (β-actin and HPRT1) was performed using TaqMan @ Gene Expression Assays on the Step One Plus real-time PCR system (Thermo Fisher Scientific Inc.) according to the manufacturer’s instructions. The assay numbers for these genes were as follows: Hs00193435_m1(HAS2), Hs00193436_m1 (HAS3), Hs00201046_m1 (HYAL1), Hs01552124_m1 (KIAA1199), Hs00154208_m1 (CA9), Hs80900055_m1 (VEGFA), Hs00194899_m1 (β-actin) and Hs02800695_m1 (HPRT1). Relative quantification was performed using Ct values to determine the expression for target genes and an internal control gene in all samples^26^.

### Western blot analysis

The cells were harvested and total protein was extracted with PRO-PREP protein extraction solution (iNtRON Biotechnology, Gyeonggi-do, South Korea), and protein concentration was determined using the bicinchoninic acid (BCA) protein assay kit (Thermo Fisher Scientific Inc., Waltham, MA, USA). Equal amounts of protein were separated on a 4–15% Mini-PROTEAN Precast Gel (Bio-Rad, Philadelphia, PA, USA) and transferred onto PVDF membranes (ATTO, Tokyo, JAPAN). Membranes were blocked for 1 h with 1% non-fat milk in TBST buffer at room temperature, then incubated with antibodies against KIAA1199 (Proteintech Group, Rosemont, IL, USA) and β-actin (Proteintech Group) overnight at 4 °C or 1 h at room temperature, followed by incubation with secondary antibodies (Proteintech Group) for 1 h at room temperature. The proteins were visualised using an ECL Western Blotting Detection System (GE Healthcare, Buckinghamshire, England)^26^. To quantify the western blotting data, the intensities of proteins of interest in each gel were firstly normalized to their respective actin intensities, then the normalized intensities were compared with the intensity of the control group and expressed as relative values to their controls^27^.

### Cell migration assay

The migratory ability of cells was determined using a transwell cell migration assay with cell culture inserts containing a filter membrane with 8 μm pores (BD Biosciences, Franklin Lajes NJ). The lower chamber was filled with RPMI1640 containing 10% foetal bovine serum (FBS). The upper chamber was filled with 1.0 × 10^4^ cells (for Panc-1) or 2.0 × 10^4^ cells (for BxPC3) in RPMI RPMI1640 containing 1% FBS. After 48 h of incubation in normoxic and 1% O_2_ conditions, the cells remaining on the upper side of the filters were removed. The cells on the bottom surface of the membrane were stained with haematoxylin and eosin, and the number of cells that had migrated to the bottom surface of the membrane were counted in five randomly selected microscopic fields for each sample^26^.

### siRNA targeting for KIAA1199

The siRNA for KIAA1199 (ON-TARGET plus SMART pool Human KIAA1199 L022291-00) and negative control siRNA (ON-TARGET plus Control siRNA Non-Targeting siRNA #1 D-001810-01-05) were purchased from GE Healthcare (Buckinghamshire, England). PDAC cells (Panc-1 and BxPC3) were transfected with 100 nM siRNA using DharmaFECT 1 Transfection Reagent (GE Healthcare) according to the manufacturer’s instructions. After 48 h of treatment, the cells were immediately used for further experiments^26^.

### Immunohistochemistry

Formalin-fixed and paraffin-embedded sections were stained for KIAA1199 (1:100; Proteintech Group, Inc., Chicago, IL, USA) and HIF1α (1:50; Novus Biologicals, Inc. Littleton, CO, USA). EnVision + System- HRP Labelled Polymer Anti-Rabbit (Dako North America, Inc., Carpenteria, CA, USA) was used as the secondary antibody for anti-KIAA1199, and stained with diaminobenzidine (Liquid DAB + Substrate Chromogen System; Dako North America, Inc.). Secondary antibody staining for anti-HIF1α was performed using the streptavidin–biotin technique using a SAB-PO(M) kit (NICHIREI BIOSCIENCES INC, Japan). Sections were counterstained with haematoxylin, dried, and mounted. The proportion of positive cells was evaluated throughout the tissue section and was graded as follows: 0 (< 11%), 1 (11–40%), 2 (41–70%), or 3 (> 70%). Staining intensity was graded as 1 (weak), 2 (moderate), or 3 (strong). The total score was obtained by multiplying the positive proportion score with the intensity score. We then divided all cases into two groups as the high expression group (with a score ranging from 3 to 9) and low expression group (with a score ranging from 0 to 2)^20^.

### Ethics statement

Written informed consent was obtained from all patients whose tissues were used in this study. The study was approved by the Institutional Review Board of the University of Occupational and Environmental Health, Kitakyushu, Japan. All methods were performed in accordance with the relevant guidelines and regulations.

### Statistical analysis

All statistical analyses were performed using STATA15 software (StataCorp. 2017. *Stata Statistical Software: Release 15.* College Station, TX: StataCorp LLC. Student’s t-test and the chi-squared test were used for group comparison. Statistical significance was set at *p* < 0.05.

## Results

### Changes in the expression of HA-synthesizing and degrading enzymes in PDAC cell lines under normoxic and hypoxic conditions

**Figure 1:**
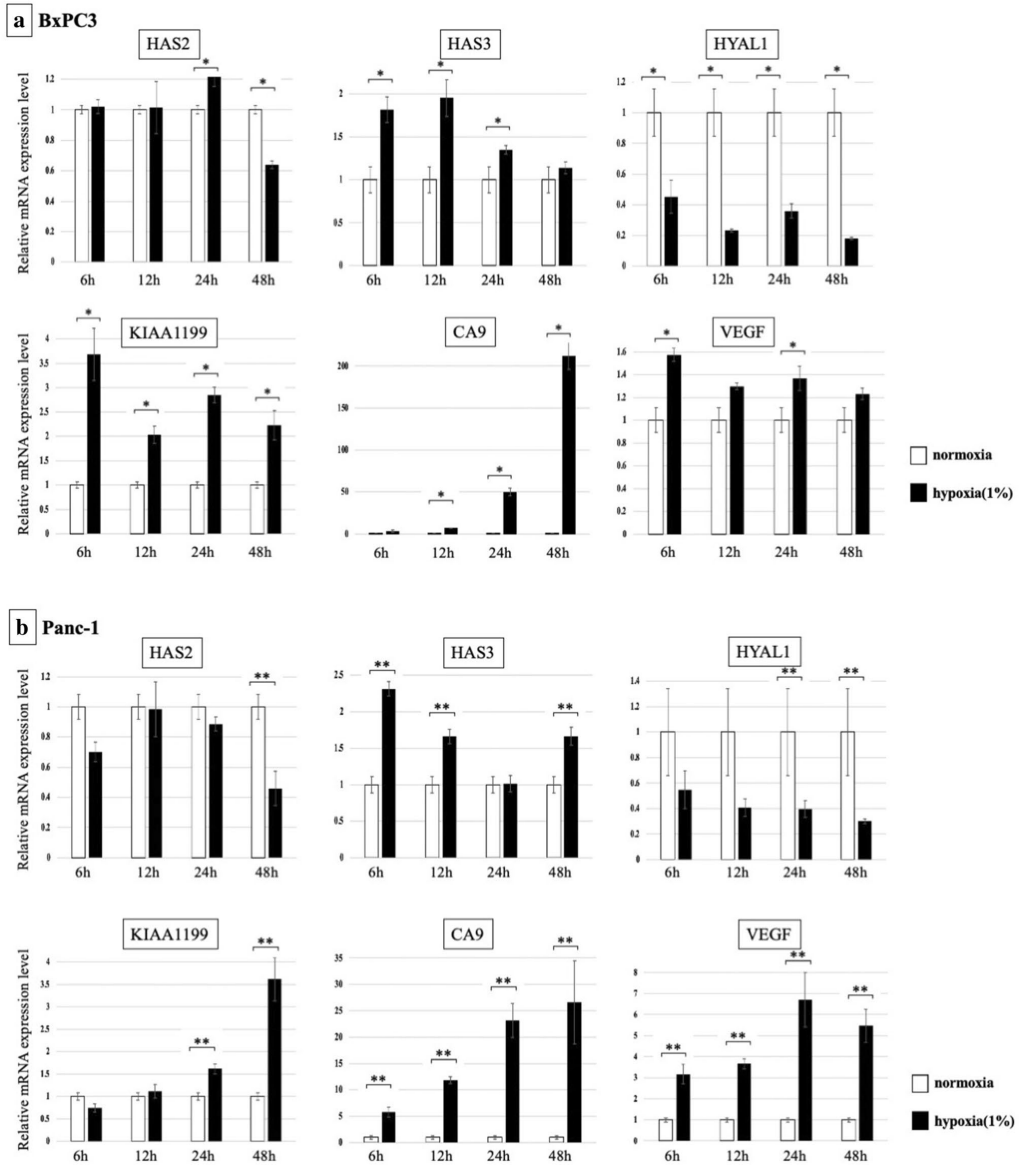
Changes in mRNA expression of HA synthesizing enzymes and HA degrading enzymes are shown. (**a**) BxPC3 cells: Expression of KIAA1199 and HAS3 was markedly increased under hypoxia, whereas HYAL1 was decreased (*p < 0.05). (**b**) Panc-1 cells: Expression of KIAA1199 was significantly increased under hypoxia from 24 to 48 h, and HYAL1 was significantly decreased. HAS3 was also enhanced under hypoxic condition (**p < 0.05).

Changes in the mRNA expression of HA-synthesizing enzymes (HAS2 and HAS3) and HA-degrading enzymes (HYAL1, KIAA1199) upon hypoxia were examined. In BxPC3 cells, KIAA1199 was markedly increased under hypoxia (> 3.5-fold increased at 6 h), whereas the expression of HYAL1 was decreased under hypoxia (< 0.5-fold decreased from 6 to 48 h). HAS3 was enhanced under hypoxia (Fig. 1a). In Panc-1 cells, the expression of KIAA1199 was also significantly increased under hypoxia from 24 to 48 h (> 3.5-fold increased at 48 h), and the expression of HYAL1 was significantly decreased (< 0.5-fold decreased from 12 to 48 h). HAS3 was also enhanced under hypoxic condition (Fig. 1b). To confirm hypoxic condition in our experiments, we examined mRNA expression of CA9 and VEGF, well-recognized HIF target. CA9 and VEGF were significantly increased in response to hypoxia in both cell lines.

**Figure 2:**
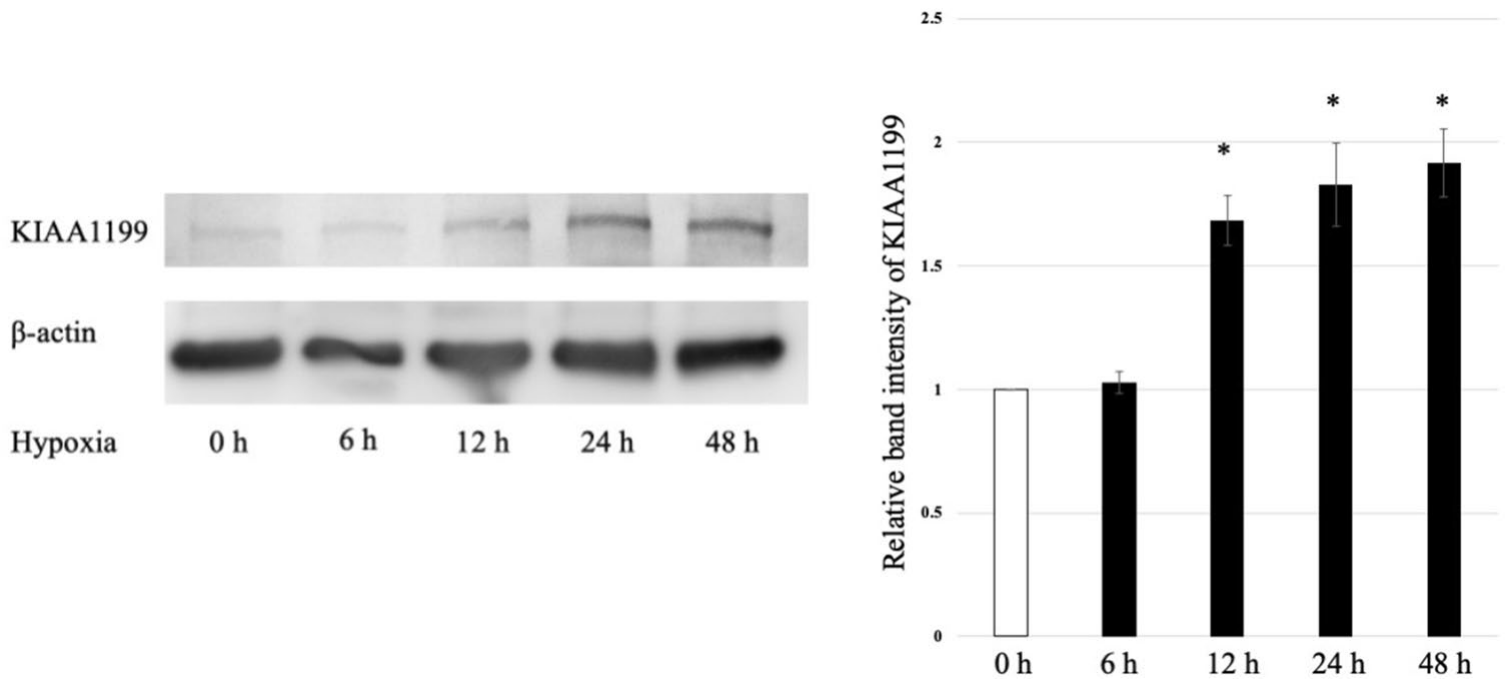
KIAA1199 protein increased in a time-dependent manner under hypoxic conditions. β-actin levels were used as loading controls. *p < 0.05 vs. normoxic condition.

The changes in the protein expression of KIAA1199 under hypoxia in BxPC3 cells was analysed using western blot analysis. KIAA1199 protein expression was increased in a time-dependent manner under hypoxic conditions (Fig. 2 and Fig.S1). β-actin levels were used as the loading controls.

### Migratory ability of PDAC cells under hypoxic conditions

**Figure 3:**
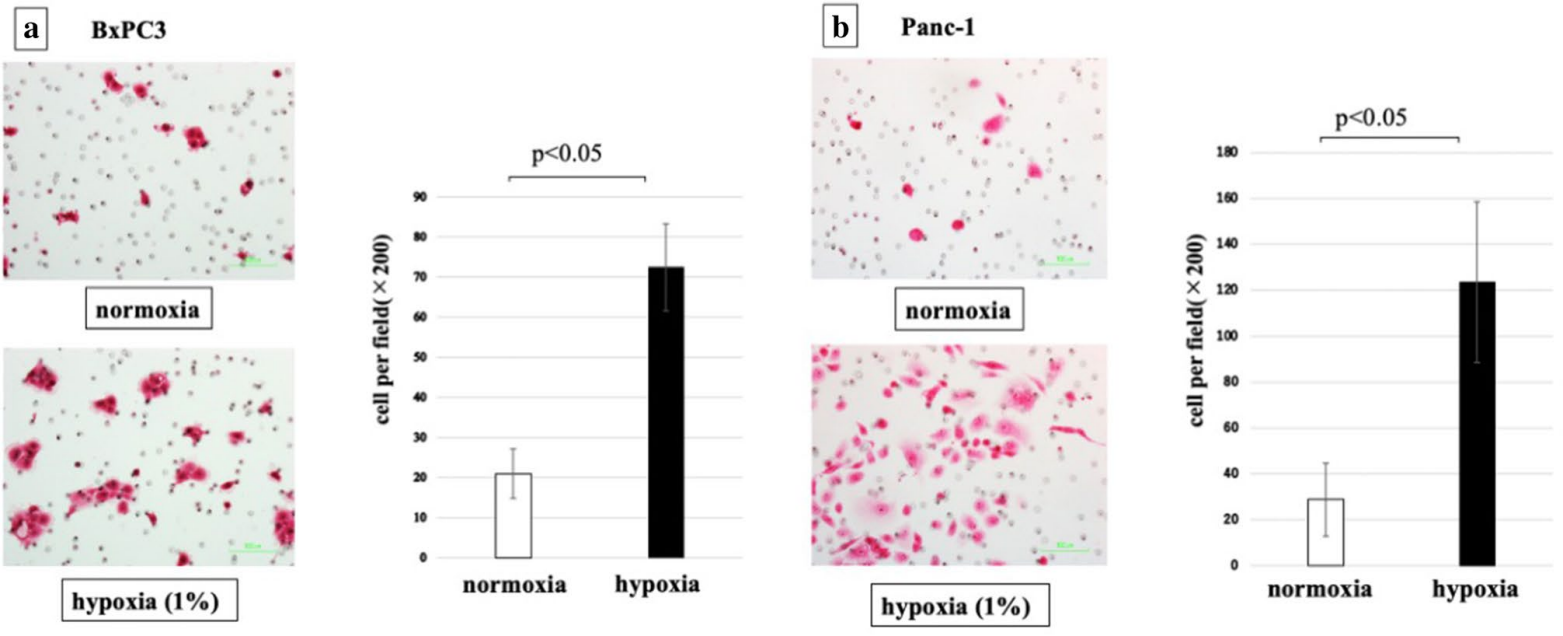
Cell migratory ability of PDAC cells was significantly increased by hypoxia (p < 0.05). (**a**) BxPC3 cells, (**b**) Panc-1 cells.

We investigated the migratory ability of PDAC cells, Panc-1 and BxPC3, under hypoxic conditions. In both cell lines, migratory ability was increased under hypoxic conditions (Fig. 3). The migratory ability of Panc-1 cells was increased up to 400% and BxPC3 cells was up to 350% under hypoxic conditions.

### Effect of KIAA1199 on PDAC cell migration in hypoxic condition

**Figure 4:**
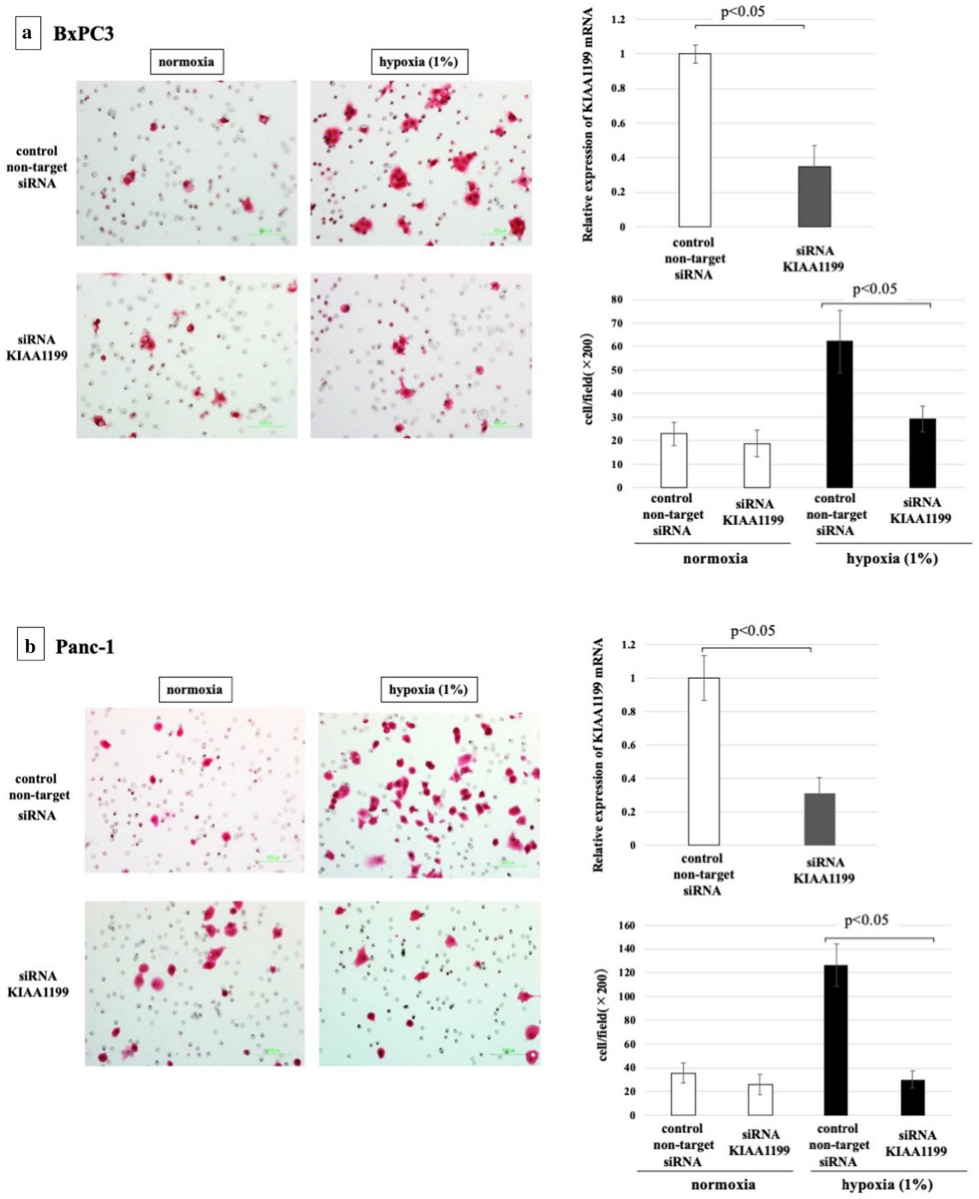
Cell migratory ability of PDAC cells under hypoxia with/without knockdown of KIAA1199. (**a**) BxPC3 cells, (**b**) Panc-1 cells. Real-time RT-PCR showed that transfection with siRNA targeting for KIAA1199 (siRNA-KIAA1199) resulted in approximately 70% knockdown*.* Knockdown of KIAA1199 inhibited the increased migration by hypoxia (p < 0.05).

Our previous studies have shown that KIAA1199 strongly stimulates the migration of PDAC cells^26^. Therefore, we hypothesised that increased expression of KIAA1199 may play a role in the enhanced migration of PDAC cells upon hypoxia. To test this, we used siRNA to knock down KIAA1199 in Panc-1 and BxPC3 cells and examined their migration under hypoxic conditions (Fig. 4). Real-time RT-PCR showed that transfection with siRNA targeting for KIAA1199 (siRNA-KIAA1199) resulted in approximately 70% knockdown*.* KIAA1199 knockdown significantly inhibited the hypoxia-induced increase in PDAC cell migration in both cell lines (*p* < 0.05).

### Immunohistochemical analysis of KIAA1199 and HIF1α in PDAC

**Figure 5:**
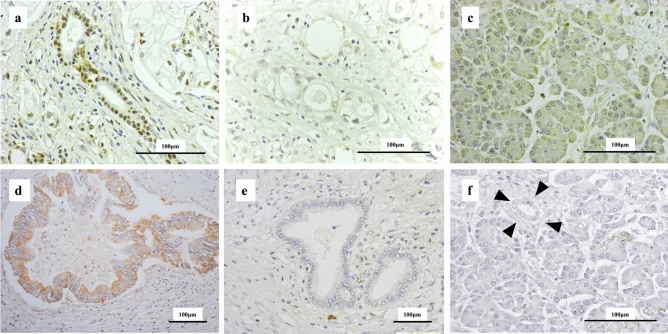
Immunostaining of PDAC tissues using anti-HIF1α antibody and anti-KIAA1199 antibody. (**a**) A representative image of strong nuclear labelling in PDAC cells (classified as high HIF1α expression group). (**b**) A representative image classified as the low HIF1α expression group. (**c**) Acinar cells with weak cytoplasmic labelling for HIF1α. (**d**) A representative image of strong cytoplasmic labelling of PDAC cells (classified as the high KIAA1199 expression group). (**e**) A representative image classified as the low KIAA1199 expression group. (**f**) Acinar cells and noncancerous duct with no labelling for KIAA1199 (arrow head).

We then examined the relationship between KIAA1199 and HIF1α expression, as a marker of hypoxia, using immunohistochemical analysis of archival tissues from 25 patients with PDAC. Representative immunostaining results are shown in Fig. 5 and Supplementary Figure S2. KIAA1199 expression was found to be high in 13 patients (52%), and HIF1α was highly expressed in 14 patients (56%). There was a significant positive correlation between KIAA1199 and HIF1α expression in our patient group (*p* < 0.05) (Table 1).

**Table 1:** Immunostaining of PDAC tissue with anti-HIF1α antibody and anti-KIAA1199 antibody.

	KIAA1199	
	High	Low
**HIF1α**		
High	10	4
Low	3	8

There was a significant positive correlation between KIAA1199 and HIF1α expression in our patient group (p < 0.05).

## Discussion

In the present study, the changes in HA metabolism in PDAC cell lines under hypoxic conditions were investigated. The major findings were as follows: (1) Hypoxia increased the mRNA and protein expression of KIAA1199 in PDAC cells. (2) Hypoxia enhanced the migration of PDAC cells, whereas knockdown of KIAA1199 inhibited the migration enhanced by hypoxia. (3) There was a significant immunohistochemically positive correlation between HIF1α and KIAA1199 in PDAC tissues.

In our previous studies, we showed that HA metabolism was accelerated in PDAC cells^10,28–30^. However, the expression patterns of HA-synthesizing or degradation enzymes under hypoxia in PDAC have been poorly characterised. In this study, we demonstrated, for the first time, that hypoxia markedly increased the expression of KIAA1199, which is known to be involved in the malignant behaviour of cancer cells, in PDAC cells. These findings suggest that changes in HA metabolism under hypoxia are associated with the malignant phenotype of PDAC cells.

Previous studies have revealed the mechanisms by which KIAA1199 promotes cancer progression. For example, KIAA1199 promotes the development of ovarian cancer by regulating PI3K/AKT signalling^31^. KIAA1199 regulates the proliferation and migration of vascular smooth muscle cells (VSMCs) in atherosclerosis by activating the Wnt-β-catenin signalling pathway^32^. Furthermore, KIAA1199 is known to increase the migration of cancer cells partly by mediating endoplasmic reticulum calcium leakage, which results in protein kinase Cα activation^33^. In our previous studies, knockdown of KIAA1199 expression was found to result in decreased migration, whereas forced expression of KIAA1199 increased the migration, invasion, and proliferation of PDAC cells^20,26^. In the present study, we also demonstrated that knockdown of KIAA1199 inhibits enhanced migration under hypoxia, suggesting the possible involvement of KIAA1199 in the aggressive phenotype under hypoxia. Our present results and the previous findings may provide a rationale for developing novel therapeutic interventions targeting KIAA1199 for treating PDAC in the future.

Our previous studies identified a novel phenotype, HAMP (hyaluronan-activated metabolism phenotype), characterized by activation of multiple HA metabolism cascades, in PDAC^34^. HAMP was defined as the increased expression of multiple HA metabolism genes, including KIAA1199. However, the mechanisms underlying the increased expression of these HA metabolism genes in PDAC are unclear. One such mechanism could be the tumour microenvironment (or tumour-stromal interactions) in PDAC. Our present results suggest that hypoxia could contribute to the development of the HAMP phenotype by increasing the expression of KIAA1199. Further studies are thus needed to elucidate the relationship between hypoxia and HAMP in PDAC.

In conclusion, our present study suggests that hypoxia-induced KIAA1199 expression may contribute to enhanced motility and thus microenvironment-mediated cancer progression in PDAC.

## Supplementary Information

Supplementary Information.

## Data Availability

The datasets generated and/or analysed during the current study are available from the corresponding author upon reasonable request.
