# Supplementary material for: Hypoxia increases KIAA1199/CEMIP expression and enhances cell migration in pancreatic cancer
Source: Sci Rep. 2021 Sep 14;11:18193. doi: 10.1038/s41598-021-97752-z (PMC8440617; doi:10.1038/s41598-021-97752-z)
Supplement: Supplementary file 1 — Supplementary Information. [file 41598_2021_97752_MOESM1_ESM.pdf]

Supplementary information file

# Hypoxia increases KIAA1199/CEMIP expression and enhances cell migration in pancreatic cancer

Takuya Oba*[1], Norihiro Sato[1], Yasuhiro Adachi[1], Takao Amaike[1], Yuzan Kudo[1], Atsuhiro Koga[2], Shiro Kohi[1], Keiji Hirata[1]

Affiliations
1) Department of Surgery 1, School of Medicine, University of Occupational and Environmental Health, Kitakyushu, JAPAN
2) Department of Surgery, Kyushu Rosai Hospital, Kitakyushu, JAPAN

Corresponding author:
Takuya Oba, MD
Department of Surgery 1, School of Medicine, University of Occupational and Environmental Health, Kitakyushu 807-8555, JAPAN
+81-93-691-7441
t.oba0205@gmail.com

Supplementary Figure.S1

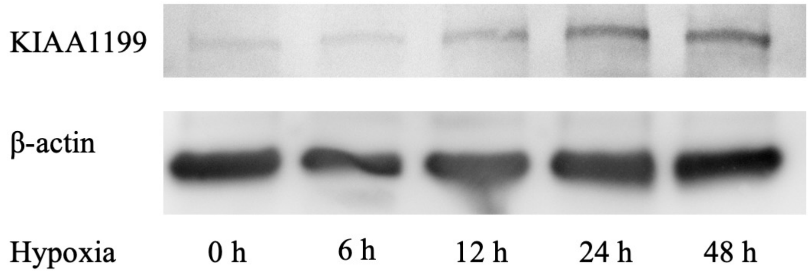


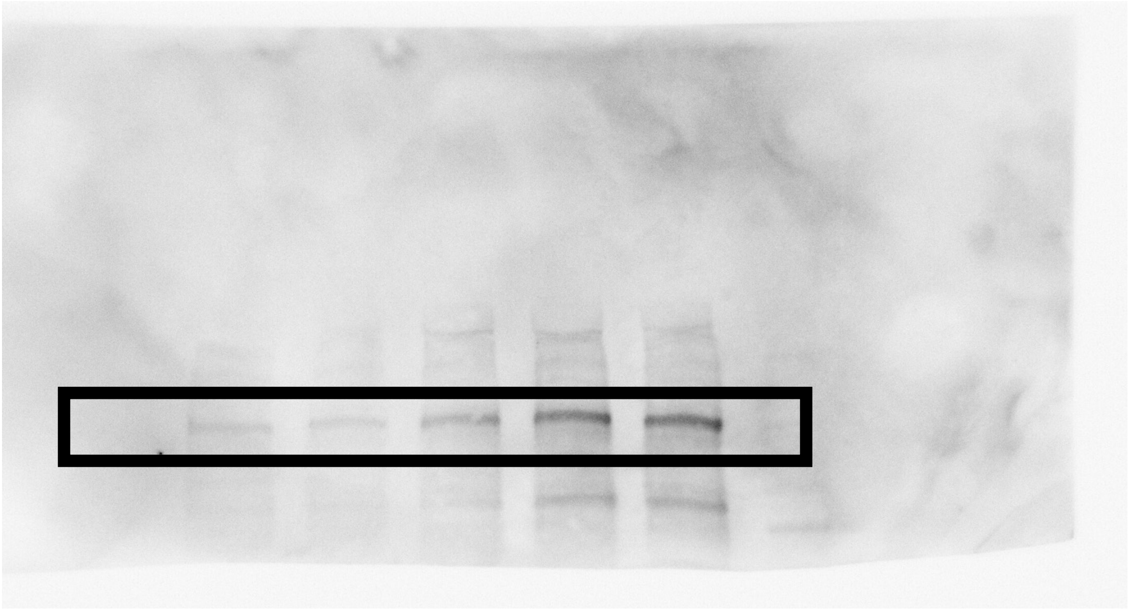

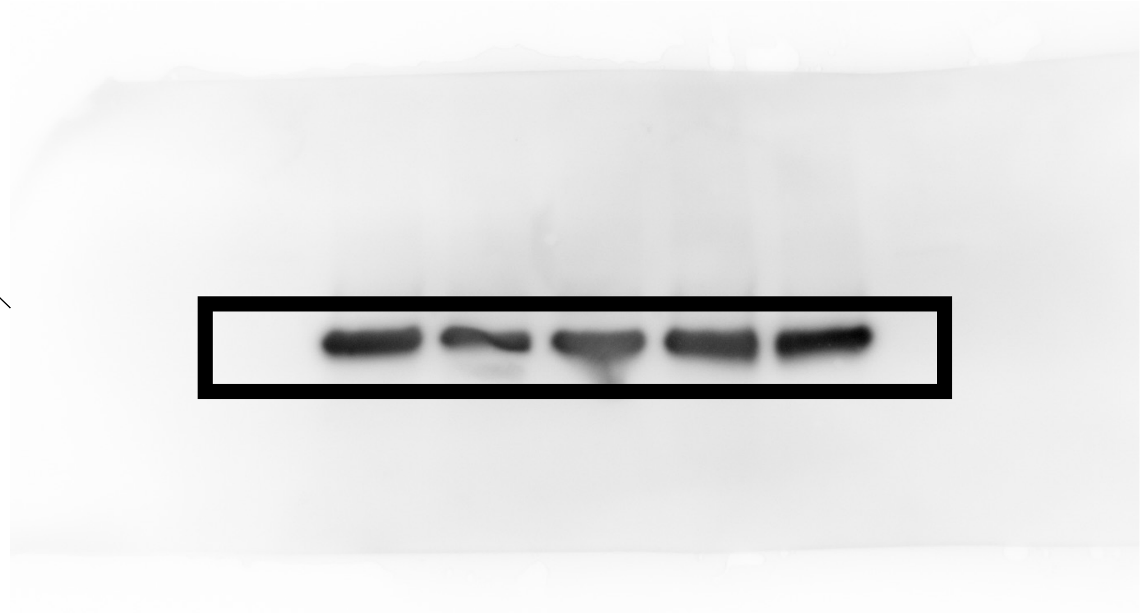

Title: Changes in expression of KIAA1199 in PDAC cell lines under normoxic and hypoxic conditions

Legend:
KIAA1199 protein increased in a time-dependent manner under hypoxic conditions. β-actin levels were used as a loading control.

Supplementary Figure.S2

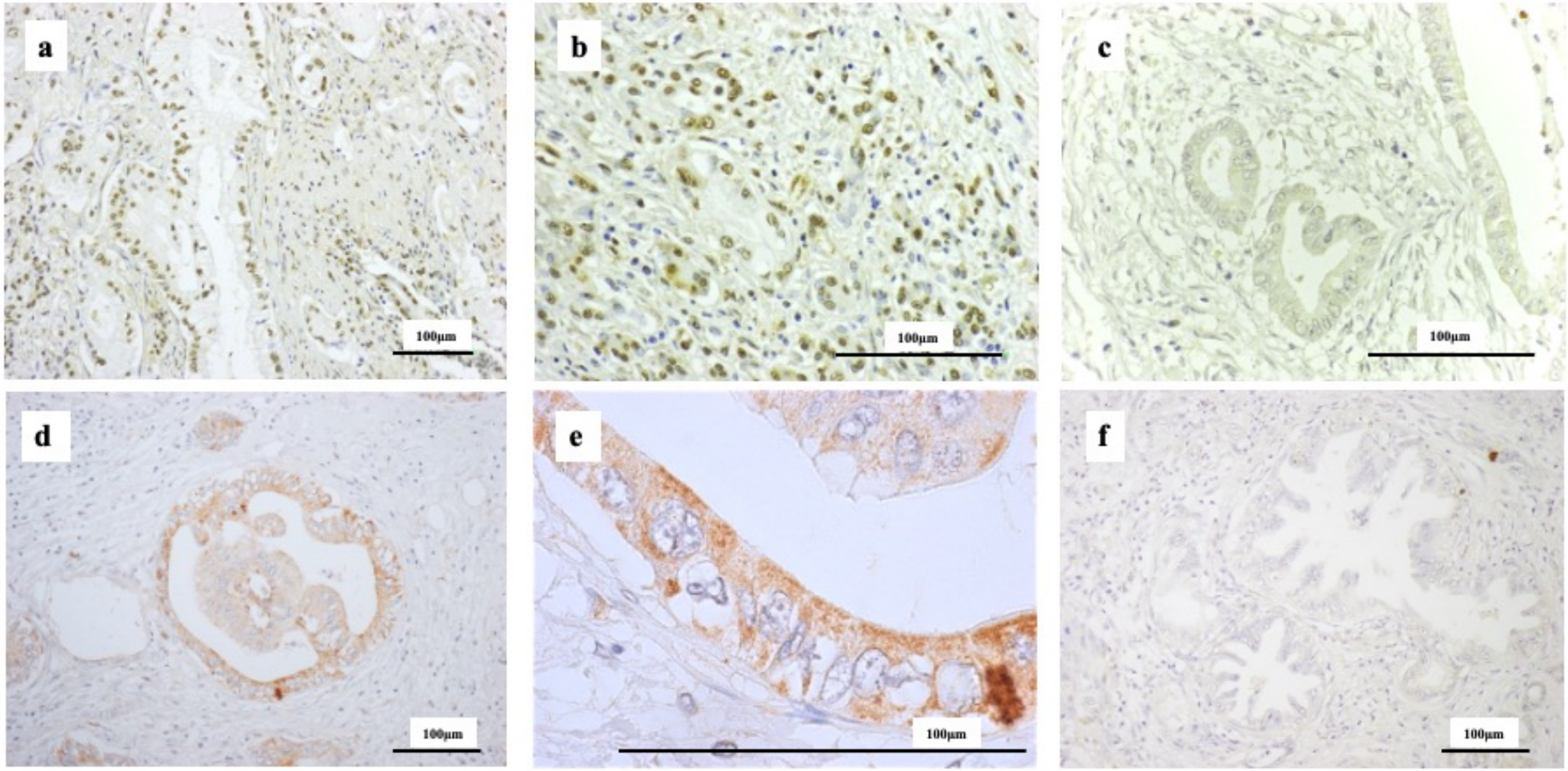


Title: Immunohistochemical analysis of KIAA1199 and HIF1α in PDAC

Legend:

(a) (b) Images of strong nuclear labelling in PDAC cells classified as high HIF1α expression group. (c) A Image as low HIF1α expression group.
(d) (e) Images of strong cytoplasmic labelling in PDAC cells classified as high KIAA1199 expression group. (f) A Image as low KIAA1199 expression group.
